# Blue Mussel-Derived Bioactive Peptides PIISVYWK (P1) and FSVVPSPK (P2): Promising Agents for Inhibiting Foam Cell Formation and Inflammation in Cardiovascular Diseases

**DOI:** 10.3390/md22100466

**Published:** 2024-10-10

**Authors:** Chathuri Kaushalya Marasinghe, Jae-Young Je

**Affiliations:** 1 Department of Food and Nutrition, Pukyong National University, Busan 48513, Republic of Korea; chathurimarasinghe9313@gmail.com; 2 Major of Human Bioconvergence, Division of Smart Healthcare, Pukyong National University, Busan 48513, Republic of Korea

**Keywords:** blue mussel, bioactive peptides, RAW264.7 macrophages, atherosclerosis, inflammation, cholesterol flux, oxidative stress

## Abstract

Atherosclerosis is a key etiological event in the development of cardiovascular diseases (CVDs), strongly linked to the formation of foam cells. This study explored the effects of two blue mussel-derived bioactive peptides (BAPs), PIISVYWK (P1) and FSVVPSPK (P2), on inhibiting foam cell formation and mitigating inflammation in oxLDL-treated RAW264.7 macrophages. Both peptides significantly suppressed intracellular lipid accumulation and cholesterol levels while promoting cholesterol efflux by downregulating cluster of differentiation 36 (CD36) and class A1 scavenger receptors (SR-A1) and upregulating ATP binding cassette subfamily A member 1 (ABCA-1) and ATP binding cassette subfamily G member 1 (ABCG-1) expressions. The increased expression of peroxisome proliferator-activated receptor-gamma (PPAR-γ) and liver X receptor-alpha (LXR-α) further validated their role in enhancing cholesterol efflux. Additionally, P1 and P2 inhibited foam cell formation in oxLDL-treated human aortic smooth muscle cells and exerted anti-inflammatory effects by reducing pro-inflammatory cytokines, nitric oxide (NO), prostaglandin E_2_ (PGE_2_), inducible nitric oxide synthase (iNOS), and cyclooxygenase-2 (COX-2), primarily through inhibiting NF-κB activation. Furthermore, P1 and P2 alleviated oxidative stress by activating the Nrf2/HO-1 pathway. Our findings demonstrate that P1 and P2 have significant potential in reducing foam cell formation and inflammation, both critical factors in atherosclerosis development. These peptides may serve as promising therapeutic agents for the prevention and treatment of CVDs associated with oxidative stress and inflammation.

## 1. Introduction

Atherosclerosis, a significant vascular pathology, has emerged as a leading cause of mortality in both Korea and Western countries [1], serving as a crucial etiological event in cardiovascular diseases (CVDs), encompassing myocardial infarction, ischemic diseases, strokes, and peripheral arterial disease [2]. The global burden of CVDs is substantial, with over 19 million reported deaths in 2019, and expected to rise to 25 million by 2030 [3]. Foam cells, a key pathological component which contribute significantly to the progression of atherosclerosis, are commonly found within atherosclerotic plaque as pathogenic cells [4]. Under normal conditions, intraplaque macrophages maintain a balanced cellular microenvironment through lipid metabolism, immune regulation and modulation, and efferocytosis. However, the recruitment of monocytes into the vascular intima and their exposure to the modified lipoproteins contribute to the formation of lipid-laden macrophage foam cells in the context of atherosclerosis [5]. These foam cells not only trigger the initial stages of atherosclerosis but also play a pivotal role in the progression of advanced plaque rupture [6].

The aggregation of altered low-density lipoproteins (LDL), particularly oxidized LDL (oxLDL), and the subsequent disruption of cholesterol metabolism are crucial factors in the ongoing presence of lipid-filled foam cells [7]. Under normal physiological circumstances, scavenger receptors facilitate lipid uptake via cholesterol influx process, while ABC transporters mediate cholesterol efflux to release excess lipid [8]. However, oxLDL disrupts these cholesterol flux processes, leading to an exacerbation of foam cell formation and its associated complications [8]. Furthermore, inflammation emerges as a fundamental contributor to the pathogenesis of atherosclerosis. Inflammatory responses are intricately involved in all stages of atherosclerosis, with oxLDL playing a major role in triggering inflammation by stimulating the generation of inflammatory factors [9]. The interplay between foam cell formation and inflammation amplifies the complexities of atherosclerosis.

Investigating natural agents with potential anti-inflammatory properties to mitigate foam cell formation presents an attractive strategy for prevention of atherosclerosis. Marine-derived organisms have garnered attention due to their perceived health benefits in preventing several chronic diseases [10]. These organisms boast a diverse array of active compounds, comprising proteins, carbohydrates, lipids, and polyphenols [11]. Of particular interest, proteins have the capacity to produce bioactive peptides (BAPs) with certain biological activities, including antioxidant, anticancer, anti-obesity, and anti-diabetes effects [12,13]. Recent studies have highlighted various BAPs with significant health benefits, particularly in the context of CVDs. For instance, BAPs derived from collagen hydrolysate of *Salmo salar* skin have been shown to reduce blood pressure and lipids [14], while pentapeptides VIAPW and IRWWW from *Miichthys miiuy* muscle exhibit hypolipidemic activities [15]. Similarly, antioxidant peptides from *Eucheuma cottonii*, a marine red alga [16], demonstrate promising cardiovascular benefits. Furthermore, antihypertensive peptides from Antarctic krill protein hydrolysates offer additional therapeutic potential [17]. The cumulative evidence suggests that these BAPs not only contribute to lowering blood pressure and lipids but also possess antioxidant and anti-inflammatory properties, making them valuable in managing heart disease and atherosclerosis.

Blue mussel (*Mytilus edulis*), a marine bivalve, is recognized as a substantial source of proteins, constituting approximately 64.5% of dry matter, along with a rich profile of amino acids [18]. Several studies, including our own, have highlighted antioxidant, hepatoprotective, anti-inflammatory, and anti-osteoporotic activities exhibited by active substances derived from blue mussel hydrolysates and isolated peptides [19,20,21,22,23]. Additionally, seventeen novel angiotensin-converting enzyme (ACE) inhibitory peptides have been identified from the protein hydrolysate of *Mytilus edulis*. These peptides demonstrate potential therapeutic value in the treatment of high blood pressure, atherosclerosis, and heart disease [24]. Moreover, in a preliminary investigation, blue mussel-derived hydrolysate demonstrated a promising inhibitory effect on foam cell formation [25]. Building upon these findings and acknowledging the multifunctional biological activities of BAPs [26], including our own findings related to osteogenesis effects, antioxidant properties, and anti-apoptosis effects of blue mussel-derived peptides, PIISVYWK (P1) and FSVVPSPK (P2), the present study delves into the foam cell formation inhibitory effects of these two peptides (P1 and P2) in oxLDL-treated RAW264.7 macrophages. Furthermore, the study scrutinizes the anti-inflammatory and anti-oxidative stress properties of these two peptides in oxLDL-treated RAW264.7 macrophages.

## 2. Results

### 2.1. Effects of P1 and P2 Peptides on Cell Viability and on oxLDL-Induced Macrophage Foam Cell Formation

**Figure 1:**
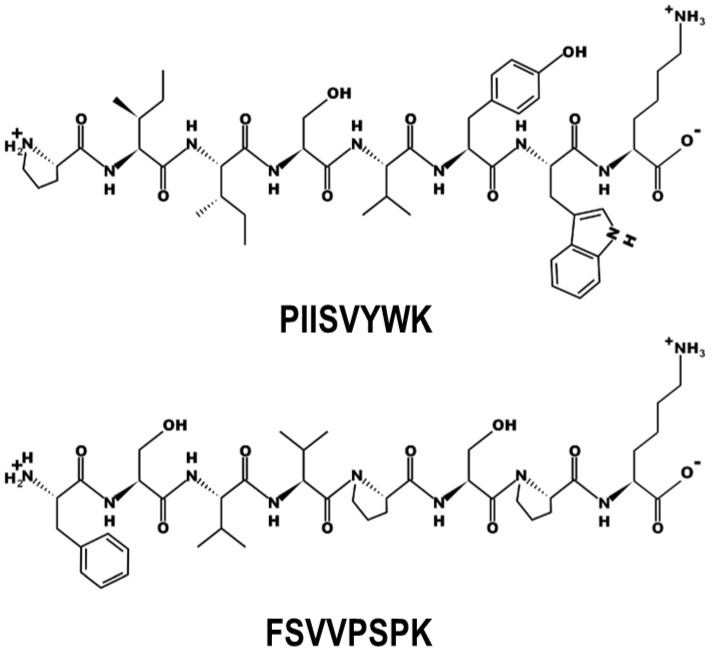
Chemical structures of PIISVYWK (P1) and FSVVPSPK (P2).

**Figure 2:**
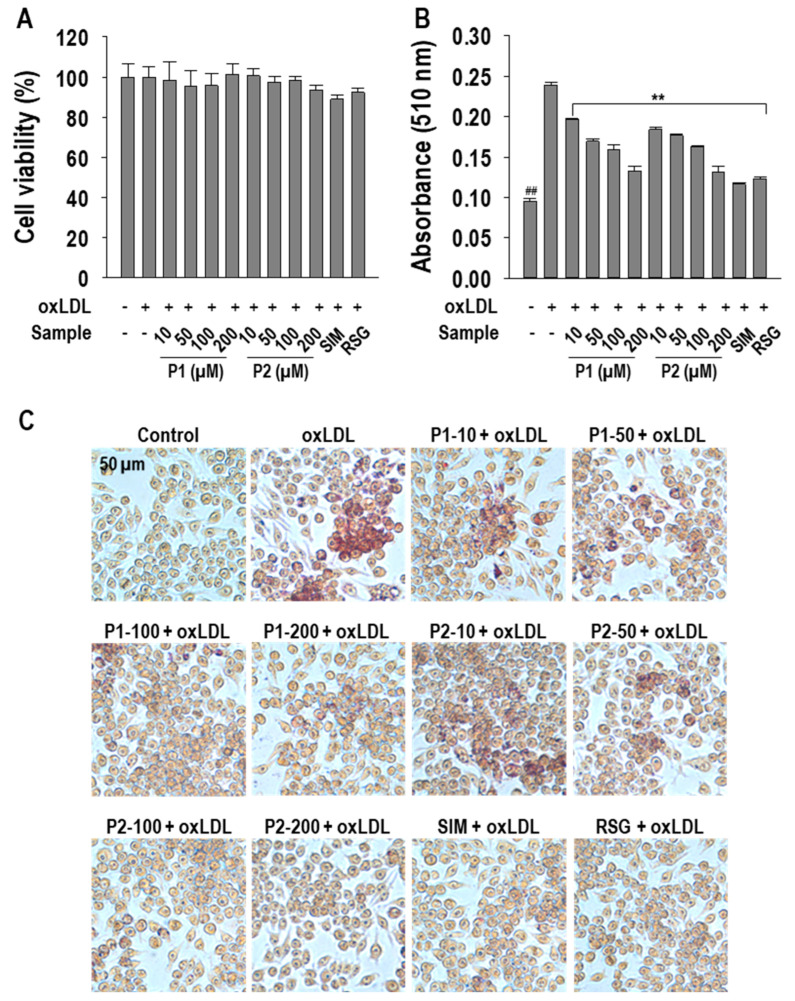
(**A**) Cell viability, (**B**) inhibition of intracellular lipid accumulation quantitatively, and (**C**) qualitatively (40× magnification) of PIISVYWK (P1) and FSVVPSPK (P2) peptides in oxLDL-treated RAW264.7 macrophages. The macrophages were treated with P1 and P2 peptides or positive controls (10 µM) including simvastatin (SIM) or rosiglitazone (RSG) or oxLDL (50 µg/mL) for MTT assay. For ORO staining, macrophages were treated with P1 and P2 peptides or positive controls (10 µM) including SIM or RSG for 1 h followed by oxLDL treatment for another 24 h. Experiments consisted of three independent measurements (*n* = 3) with ± S.D. ** *p* < 0.001 and ^##^ *p* < 0.001, compared to the oxLDL-treated group and non-treated group, respectively. The numbers behind letters in images denote concentrations in µM.

The chemical structures of P1 and P2 peptides are depicted in Figure 1. Prior to investigating the inhibitory effects of P1 and P2 peptides on foam cell formation, the cytotoxicity of P1 and P2 peptides was measured in RAW264.7 macrophages. As illustrated in Figure 2A, there were no discernible cytotoxic effects at the concentrations tested (10~200 µM). To validate foam cell formation inhibitory activity, Oil Red O (ORO) staining assay was performed. Both P1 and P2 peptides demonstrated a dose-dependent inhibitory effect on macrophage foam cell formation induced by oxLDL exposure. Interestingly, P1 and P2 peptides showed similar intracellular lipid accumulation inhibition rates of approximately 74.0 ± 4.3% and 75.1 ± 5.2%, respectively, at 200 µM treatment (Figure 2B). Representative images further verify the inhibitory effect of P1 and P2 peptides (Figure 2C).

### 2.2. Effects of P1 and P2 Peptides on Total Cholesterol (TC), Free Cholesterol (FC), and Cholesterol Esters (CE), and Triglyceride (TG) Levels in oxLDL-Treated Macrophages

**Figure 3:**
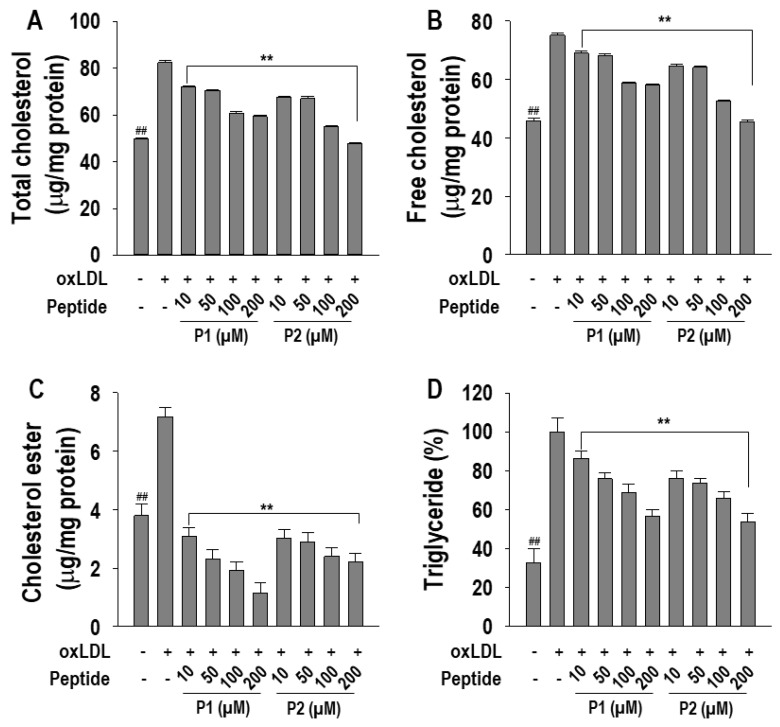
Effect of 10~200 µM of P1 and P2 peptides on (**A**) total cholesterol, (**B**) free cholesterol, (**C**) cholesterol ester, and (**D**) triglycerides content in oxLDL-treated RAW264.7 macrophages. The macrophages were treated with P1 and P2 peptides for 1 h followed by oxLDL treatment for 24 h. Experiments consisted of three independent measurements (*n* = 3) with ± S.D. ** *p* < 0.001 and ^##^ *p* < 0.001, compared to the oxLDL-treated group and non-treated group, respectively.

The effects of P1 and P2 peptides on changes in TC, FC, and CE levels were analyzed in oxLDL-treated RAW264.7 macrophages. As shown in Figure 3A~D, both P1 and P2 peptides exhibited a dose-dependent suppression of TC, FC, and CE levels. However, at a concentration of 200 µM, P2 displayed a more suppressive effect on TC and FC levels compared to P1 by fully suppressing cholesterol levels. P1 treatment attenuated TC and FC by 70.7 ± 1.0% and 58.3 ± 0.8%, respectively. Additionally, both P1 and P2 peptides fully inhibited CE levels at all the tested concentrations. Moreover, P1 and P2 peptides significantly (*p* < 0.05) ameliorated TG level compared to oxLDL-treated macrophages.

### 2.3. Effects of P1 and P2 Peptides on Cellular Cholesterol Flux

**Figure 4:**
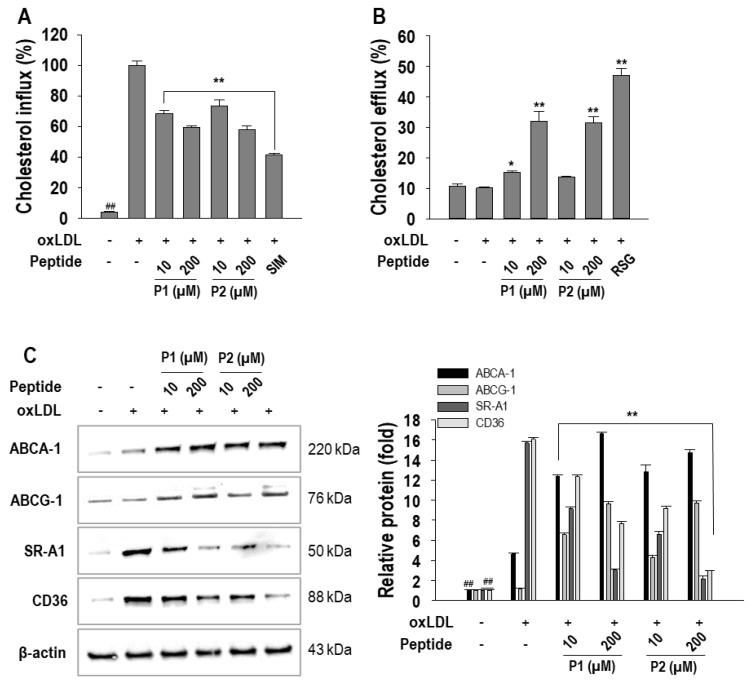
Effect of 10~200 µM of P1 and P2 peptides on (**A**) cholesterol influx, (**B**) cholesterol efflux, (**C**) ABCA-1, ABCG-1, SR-A1, and CD36 protein expressions in oxLDL-treated RAW264.7 macrophages. The macrophages were treated with P1 and P2 peptides for 1 h followed by oxLDL treatment for 24 h. Experiments consisted of three independent measurements (*n* = 3) with ± S.D. ** *p* < 0.001 and ^##^ *p* < 0.001, compared to the oxLDL-treated group and non-treated group, respectively. * p < 0.05 compared to the oxLDL-treated group.

To elucidate how P1 and P2 peptides affect cellular cholesterol flux, various parameters including cholesterol influx, cholesterol efflux, expression of relevant proteins, and transcription factor were examined. Since 10, 50, 100, and 200 µM of concentrations showed significant effect on above experiments, for subsequent studies, we have selected 10 µM as the lowest concentration and 200 µM as the highest concentration to further investigate the effects of these peptides. A significant increase (*p* < 0.05) in cholesterol influx rate was observed in oxLDL-treated macrophages. However, P1 and P2 peptides significantly (*p* < 0.05) reduced the rate of cholesterol influx compared to the cells with oxLDL alone. Notably, their inhibition rates were 42.2 ± 1.1% and 43.6 ± 2.2% for P1 and P2 peptides, respectively, at 200 µM treatment (Figure 4A). Simvastatin, a positive control, was shown to inhibit cholesterol influx by 61.0 ± 1.0%. Conversely, the rate of cholesterol efflux by P1 and P2 peptides significantly (*p* < 0.05) increased in a dose-dependent manner (Figure 4B). Cholesterol efflux rate was increased more than 3-fold when treated with 200 µM of P1 and P2 peptides compared to the macrophages without treatment. However, there is no change in cholesterol efflux rate in oxLDL-treated macrophages. Rosiglitazone, a positive control, showed a marked increase in cholesterol efflux rate.

**Figure 5:**
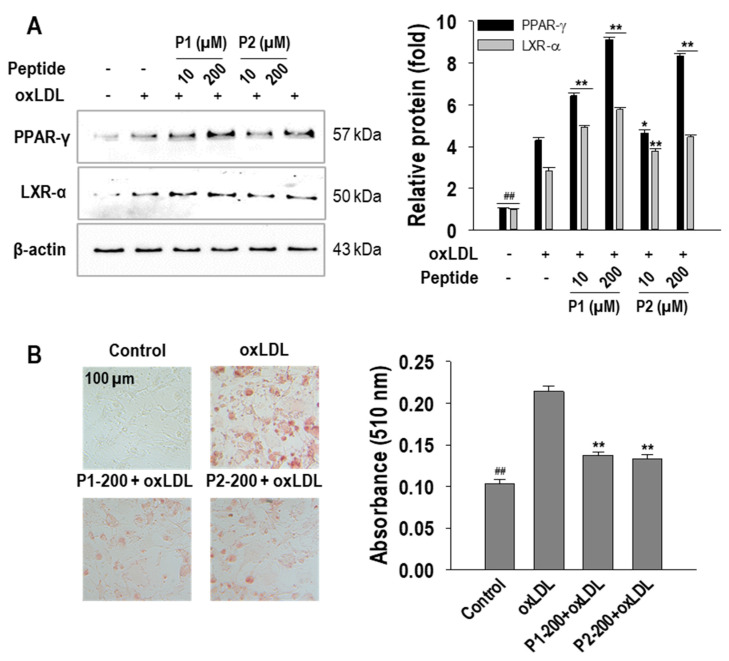
Effect of 10~200 µM of P1 and P2 peptides on (**A**) PPAR-γ and LXR-α protein expressions in oxLDL-treated RAW264.7 macrophages and (**B**) foam cell formation inhibition in hASMCs. The macrophages and hASMCs were treated with P1 and P2 peptides for 1 h followed by oxLDL treatment for 24 h. Relevant images are taken at 20× magnification. Experiments consisted of three independent measurements (*n* = 3) with ± S.D. ** *p* < 0.001 and ^##^ *p* < 0.001, compared to the oxLDL-treated group and non-treated group, respectively. * *p* < 0.05 compared to the oxLDL-treated group. In images, numbers behind letters represent concentrations in µM.

To further validate the role of P1 and P2 peptides in cholesterol flux, protein expressions associated with cholesterol influx and efflux were investigated. As shown in Figure 4C, the protein expression of ABCA-1 and ABCG-1, integral to cholesterol efflux, was elevated by P1 and P2 peptides. On the contrary, the levels of SR-A1 and CD36, which are associated with cholesterol influx, were dose-dependently suppressed by P1 and P2 peptides. In addition, the effects of P1 and P2 peptides on PPAR-γ and LXR-α protein expression, which are associated with cholesterol metabolism and flux, were investigated. Both P1 and P2 peptides significantly enhanced the expression of PPAR-γ and LXR-α. At a concentration of 200 µM, P1 treatment resulted in a 4.8 ± 0.1-fold increase in PPAR-γ expression and 2.9 ± 0.1-fold increase in LXR-α expression. Similarly, P2 treatment exhibited an increase in these transcription factor expressions, with 4.1 ± 0.1-fold enhancement in PPAR-γ expression and 1.6 ± 0.1-fold of enhancement of LXR-α expression at 200 µM concentration (Figure 5A).

Furthermore, this study extended its investigation into the foam cell formation inhibitory activities of P1 and P2 peptides using human models, especially human aortic smooth muscle cells (hASMCs). As depicted in Figure 5B, oxLDL induced a progressive increase in intracellular lipid accumulation in hASMCs. However, consistent with expectations, both P1 and P2 peptides effectively inhibited this lipid accumulation, achieving inhibition rates of approximately 69.1 ± 3.9% and 72.5 ± 4.3%, respectively. These findings underscore the potential of P1 and P2 peptides in mitigating foam cell formation not only in murine cells but also in human cells.

### 2.4. Effect of P1 and P2 Peptides on oxLDL-Induced Inflammation

**Figure 6:**
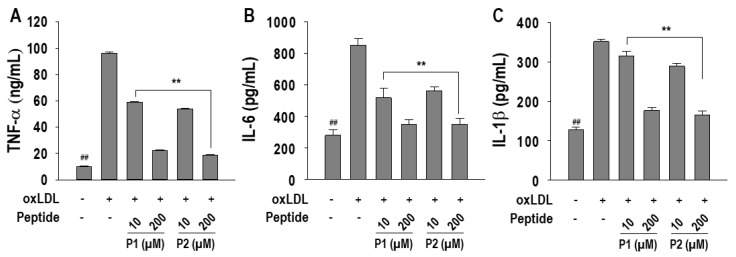
Effect of P1 and P2 peptides on pro-inflammatory cytokine production (**A**) TNF-α, (**B**) IL-6, and (**C**) IL-1β in oxLDL-treated RAW264.7 macrophages. The macrophages were treated with P1 and P2 peptides for 1 h followed by oxLDL treatment for 24 h. Experiments consisted of three independent measurements (*n* = 3) with ± S.D. ** *p* < 0.001 and ^##^ *p* < 0.001, compared to the oxLDL-treated group and non-treated group, respectively.

Given the association of atherosclerosis with inflammatory responses, this study delved into the anti-inflammatory effect of P1 and P2 peptides. Pro-inflammatory cytokine levels, including TNF-α, IL-6, and IL-1β, were assessed following treatment with P1 and P2 peptides. OxLDL treatment induced higher production of these three pro-inflammatory cytokines, and both P1 and P2 peptides significantly (*p* < 0.05) suppressed their levels (Figure 6A~C). Especially, at a concentration of 200 µM, P1 treatment inhibited TNF-α, IL-6, and IL-1β levels by 86.1 ± 0.8%, 87.9 ± 5.0%, and 78.2 ± 3.2%, respectively. Similarly, P2 treatment exhibited inhibition levels of 90.3 ± 0.7%, 87.9 ± 6.6%, and 83.1 ± 4.5% for TNF-α, IL-6, and IL-1β, respectively.

**Figure 7:**
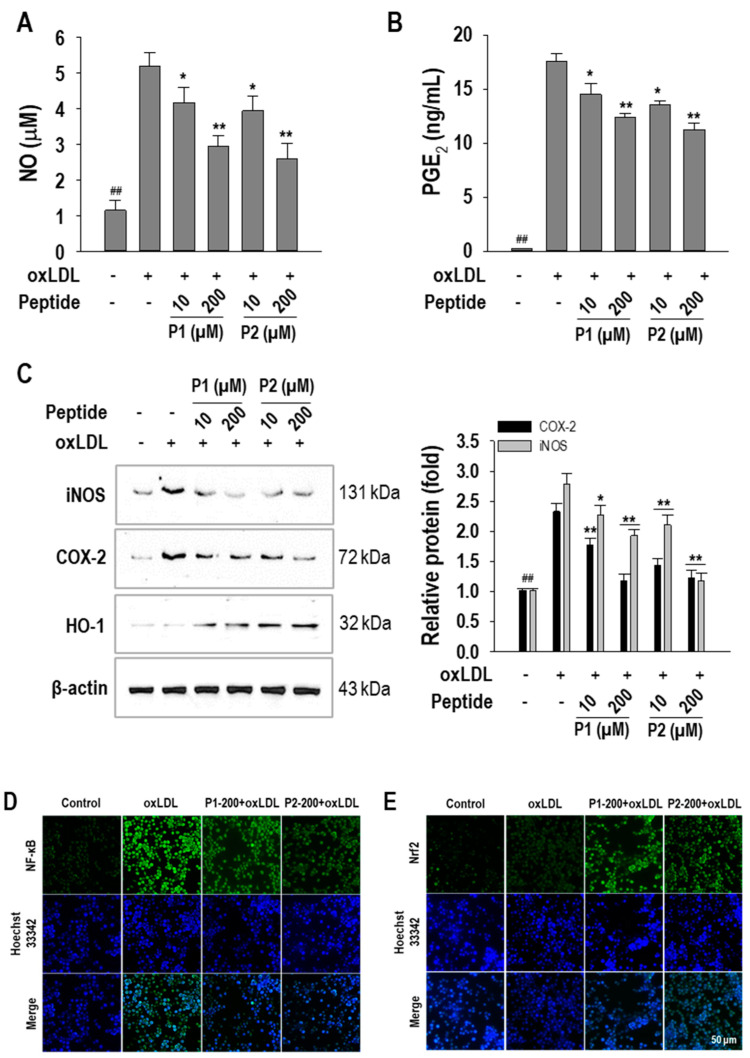
Effect of P1 and P2 peptides on (**A**) NO production, (**B**) PGE_2_ production, (**C**) iNOS, COX-2, and HO-1 protein expression in oxLDL-treated RAW264.7 macrophages. The macrophages were treated with P1 and P2 peptides for 1 h followed by oxLDL treatment for 24 h. Experiments consisted of three independent measurements (*n* = 3) with ± S.D. ** *p* < 0.001 and ^##^ *p* < 0.001, compared to the oxLDL-treated group and non-treated group, respectively. * *p* < 0.05 compared to the oxLDL-treated group. (**D**) NF-κB nuclear activation and (**E**) Nrf2 nuclear translocation in oxLDL-treated RAW264.7 macrophages. The macrophages were treated with peptides for 1 h followed by oxLDL (50 µg/mL) treatment for 2 h for NF-κB and Nrf2 immunostaining. The numbers behind letters in images denote concentrations in µM.

Moreover, NO and PGE_2_ levels, which are elevated in atherosclerosis and contribute to pro-inflammatory processes, were measured in culture media after treatment with P1 and P2 peptides. Both peptides significantly (*p* < 0.05) suppressed oxLDL-induced NO and PGE_2_ levels. At a concentration of 200 µM, P1 and P2 peptides suppressed NO levels by 55.5 ± 7.2% and 64.5 ± 11.0%, respectively (Figure 7A). Additionally, P1 and P2 peptides decreased PGE_2_ levels by 29.8 ± 2.0% and 36.4 ± 3.3%, respectively (Figure 7B). This study further investigated iNOS and COX-2 expression, which are linked to the production of NO and PGE_2_. As shown in Figure 7C, both peptides exhibited a dose-dependent suppressive effect on iNOS and COX-2 expressions. In addition, further analysis revealed that both peptides increased HO-1 expression. Finally, the effects of P1 and P2 peptides on the nuclear translocation of NF-κB and Nrf2, transcription factors for iNOS and COX-2 and HO-1, were investigated. As shown in Figure 7D, immunostaining results demonstrated that macrophages treated with oxLDL alone exhibited strong fluorescence intensity for NF-κB in the cytoplasmic and the nuclear regions, indicating inflammatory response. However, the fluorescence intensity w P1 and P2 peptides was dramatically reduced in both cytoplasmic and nuclear regions, indicating anti-inflammatory action. In addition, Nrf2 translocation was not observed in macrophages treated with oxLDL alone. However, P1 and P2 peptide-treated macrophages exhibited strong fluorescence intensity, indicating Nrf2 translocation (Figure 7E).

## 3. Discussion

Scientific evidence consistently shows that BAPs from marine sources have various biological activities, including antioxidant, anti-inflammatory, anti-cancer, and anti-diabetic properties, due to their unique environmental origins [13,27]. Despite this wealth of evidence, there is still a significant gap in research on the effects of marine BAPs in inhibiting macrophage foam cell formation and their potential athero-protective benefits. In a recent contribution to this developing field, our research team recently identified the foam cell formation inhibitory and anti-inflammatory activities of blue mussel hydrolysate in oxLDL-treated RAW264.7 macrophages [25]. Building on previous research that highlighted the anti-apoptotic, antioxidant, and anti-osteoporotic effects of blue mussel-derived P1 and P2 peptides [28,29,30], this study explores their novel role in inhibiting foam cell formation and their anti-inflammatory and anti-oxidative stress activities in oxLDL-treated RAW264.7 macrophages. The aim is to further understand the potential therapeutic benefits of these marine-derived peptides for atherosclerosis and related metabolic diseases.

Foam cell formation is recognized as a pivotal step in the pathogenesis of atherosclerosis, driven by excessive oxLDL, which leads macrophages to become foam cells [6]. Even in the early stages of atherosclerosis, many macrophages engulf oxLDL and adopt a foam cell phenotype. The vascular accumulation of these foam cells leads to arterial narrowing, further promoting the advancement of atherosclerosis [31]. Consequently, inhibiting foam cell formation is vital for combating the disease. Several studies, including our own, have reported anti-atherosclerotic effects achieved through the inhibition of foam cell formation [32,33,34]. Thus, as the primary focus in this study, we investigated the effect of P1 and P2 peptides on inhibiting foam cell formation in oxLDL-treated RAW264.7 macrophages. Consistent with our previous findings with blue mussel hydrolysates [25], the blue mussel-derived P1 and P2 peptides exhibited foam cell formation inhibitory activities, thereby indicating their anti-atherosclerotic effects.

Statin drugs, including atorvastatin, rosuvastatin, simvastatin, pravastatin, fluvastatin, lovastatin, and pitavastatin, are commonly used to lower cholesterol and treat CVDs. However, finding an accurate positive control for assessing foam cell formation inhibition has been difficult. In this study, simvastatin and rosiglitazone were chosen as positive controls, based on evidence from previous research [35,36,37]. Notably, peptides P1 and P2 demonstrated approximately equivalent levels of intracellular cholesterol inhibition compared to simvastatin and rosiglitazone, highlighting their potential in inhibiting foam cell formation.

Scavenger receptors, such as SR-A1 and CD36, are integral in the internalization of oxLDL by macrophages, leading to cellular cholesterol accumulation. Once oxLDL is internalized, oxLDL is broken down by liposomal acid lipase into free fatty acids and cholesterol [38]. To maintain cellular homeostasis, excess cholesterol is removed via ATP-binding cassettes transporters as ABCA-1 and ABCG-1 in macrophages, facilitating cholesterol efflux to Apo-A1 and HDL, respectively [8]. However, disruption in this process, with excessive cholesterol uptake and impaired efflux, causes abnormal cholesterol trafficking and foam cell formation. Research indicates that reducing cholesterol influx, while increasing cholesterol efflux, helps inhibit foam cell formation [39,40,41]. Building upon these insights, our study assessed the effects of P1 and P2 peptides on these mechanisms and found that they increased cholesterol efflux, decreased cholesterol influx, and reduced TC, FC, and CE in cells. However, the decrease in cholesterol influx and the increase in cholesterol efflux are relatively lower compared to the respective positive controls. Additionally, TG, TG-rich lipoproteins (TRL), and TRL remnants are recognized risk factors for cardiovascular diseases (CVDs) [42,43]. Interestingly, peptides P1 and P2 also inhibited TG levels, suggesting that they may contribute to the regulation of both cholesterol and lipid homeostasis.

In light of the higher rates of cholesterol efflux observed with P1 and P2 peptides compared to cholesterol influx, this study further extended to elucidating the molecular mechanism underlying cholesterol efflux. Building on previous findings where ark shell-derived peptides inhibited foam cell formation via the PPAR-γ/LXR-α signaling pathway [44], we focused on the activation of these transcription factors by P1 and P2 peptides. PPAR-γ, a nuclear receptor that forms heterodimers with Retinoid X receptor, regulates lipid metabolism genes and is a known therapeutic target for lipid disorders [45]. Similarly, LXR-α has been identified as a potential treatment for atherosclerosis [46]. However, PPAR-γ has a dual role in cholesterol metabolism: it can increase cholesterol influx by promoting CD36 expression and simultaneously enhance cholesterol efflux by upregulating ABC transporters. Despite this paradox, PPAR-γ generally reduces foam cell formation by promoting cholesterol efflux [47]. Our study found that P1 and P2 peptides increased the protein expression of both PPAR-γ and LXR-α, indicating their role in regulating lipid metabolism. Further extending our research to hASMCs, which are prominent in atherosclerotic lesions, we observed that P1 and P2 peptides reduced oxLDL-induced lipid accumulation. These results highlight the anti-atherogenic potential of P1 and P2 peptides in both murine and human cells by inhibiting cholesterol accumulation and foam cell formation.

Foam cells are linked with chronic inflammation observed in certain metabolic and autoimmune disorders. The formation of foam cells establishes a connection with inflammatory processes [48]. In particular, oxLDL induces a pro-inflammatory phenotype in macrophages, contributing to atherosclerosis [49]. Given the recognized correlation between foam cell formation and inflammation in atherosclerosis, this study aimed to clarify the role of P1 and P2 peptides in inflammatory responses. Studies have reported that oxLDL internalization leads to elevated levels of pro-inflammatory cytokines and inflammatory molecules [50]. In line with these findings, our study found that oxLDL treatment increased inflammatory responses. However, P1 and P2 peptides significantly reduced oxLDL-induced pro-inflammatory cytokines, NO, and PGE_2_ production, demonstrating their anti-inflammatory effects. Additionally, the peptides inhibited the expression of iNOS and COX-2, which are responsible for producing NO and PGE_2_, respectively.

**Figure 8:**
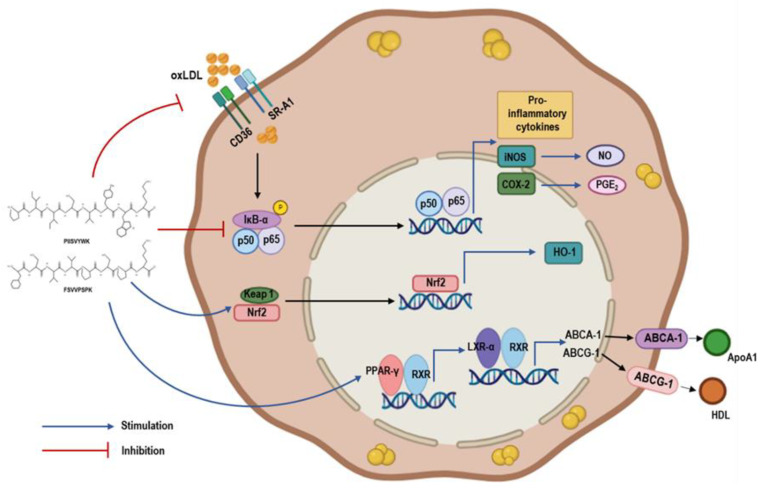
Schematic diagram for proposed mechanism.

Based on these findings, this study explored the molecular mechanisms of P1 and P2 peptides in anti-inflammation, focusing on the NF-κB pathway, which plays a key role in atherosclerosis and inflammation. Normally, NF-κB is kept inactive in the cytoplasm by binding to IκB proteins. However, inflammatory stimuli trigger the degradation of IKBα, allowing NF-κB to move to the nucleus and promote pro-inflammatory cytokines and proteins such as iNOS and COX-2 [51]. Inhibiting NF-κB signaling has been shown to reduce foam cell formation and inflammation [52]. This study found that P1 and P2 peptides inhibit NF-κB signaling in oxLDL-treated macrophages, potentially reducing inflammation and foam cell formation. Additionally, since P1 and P2 peptides increase HO-1 expression, we also examined their effect on Nrf2 activation. Nrf2, which is normally bound to Keap1 in the cytoplasm, dissociates and moves to the nucleus under oxidative stress, where it activates genes like HO-1 that have antioxidant and anti-inflammatory roles [53]. Our results showed that P1 and P2 peptides enhance Nrf2 nuclear translocation, suggesting their potential in modulating oxidative stress and inflammation in atherosclerosis. (Figure 8).

The biological activities of peptides are intricately associated with their amino acid compositions. Certain amino acids including glutamic acid, leucine, glycine, proline, lysine, and arginine have been recognized for their antiatherogenic properties [54,55,56]. P1 and P2 peptides, which share these amino acids, likely benefit from these compositions, contributing to their observed anti-atherosclerotic and anti-inflammatory activities.

## 4. Materials and Methods

### 4.1. Materials

PIISVYWK (P1) and FSVVPSPK (P2) peptides were chemically synthesized by Peptron Inc. (Dae-jeon, Korea) based on the sequences identified in our previous study [16]. The synthesized peptides were confirmed to have >95% purity, with molecular weights of 1005.2 for P1 and 860.05 for P2, respectively. Human plasma LDL was purchased from LEE BioSolutions (360-10, Lee BioSolution, Maryland Heights, MO, USA). Gibco BRL (Grand Island, NY, USA) provided all cell culture reagents. Primary and secondary antibodies were obtained from Santa Cruz Biotechnology Inc. (Santa Cruz, CA, USA), NovusBio ^®^, Colorada, USA, and Abcam (Dawinbio Inc, Seoul, Korea). All other reagents employed in this study were obtained from Sigma-Aldrich (St. Louis, MO, USA).

### 4.2. Oxidation of LDL and Determination of Thiobarbituric Acid-Reactive Substances (TBARS)

To induce oxidation, LDL (1 mg/mL) was oxidized with CuSO_4_ (10 μM) for 4 h at 37 °C, and the extent of oxidation was assessed by conducting TBARS assay according to our previous study [25]. In brief, the resulting oxLDL was mixed with TCA (25% *w*/*v*) and TBA (1% *w*/*v* in 0.3% NaOH) in equal parts, then boiled at 92 °C for 40 min in the dark. The absorbance was measured at 532 nm with a microplate reader (Multiskan™ GO, Thermo Fisher Scientific, Rockford, IL, USA). The degree of LDL modification was quantified using a standard curve of malondialdehyde (MDA), expressed as nanomoles of MDA per milligram of LDL protein. For the experiments, TBARS values ranging from 165 to 200 nM/MDA were utilized.

### 4.3. Cell Culture and Treatment

RAW264.7 macrophages (American Type Culture Collection, ATCC, Manassas, VA, USA) were cultured in a DMEM culture medium under standard conditions. Cell densities of 1 × 10^5^ or 1 × 10^6^ cells/mL were utilized for 96-well plates or 6 cm culture dishes, respectively. Primary human aorta vascular smooth muscle cells (HASMC) were procured from ScienCell Research Laboratories (Carlsbad, CA, USA) and cultured in smooth muscle cell medium (SMCM, 1101, ScienCell Research Laboratories) under standard conditions. Macrophages or hASMCs were treated with P1 or P2 for 1 h followed by exposure to oxLDL for an additional 24 h.

### 4.4. MTT Assay

Cytotoxicity of P1 and P2 in RAW264.7 macrophages and HASMC was evaluated using MTT assay according to our previous study [25]. In brief, cells were treated with P1 and P2 (10~200 µM) for 24 h, followed by a 4 h incubation with MTT working reagent. Once the formazan crystals formed, DMSO was added to dissolve them and the absorbance was measured at 570 nm using a microplate reader (Multiskan™ GO, Thermo Scientific™, Waltham, MA, USA).

### 4.5. Determination of Intracellular Lipid Accumulation Using ORO Staining Assay

RAW264.7 macrophages or HASMCs were exposed to P1 or P2 (10~200 µM) as detailed above, and ORO staining was performed to determine the effect of intracellular lipid accumulation following the protocol in our previous study [25]. 4% paraformaldehyde was added to each well for 1 h to fix the cells, followed by washing with 60% isopropanol. The cells were then stained with a freshly prepared filtered ORO solution for 1 h and washed twice with distilled water. Images were captured using an inverted microscope (DMI6000, Leica, Wetzlar, Germany) and quantification was performed by measuring absorbance at 510 nm with a microplate reader (Multiskan™ GO, Thermo Scientific™, Waltham, MA, USA).

### 4.6. Determination of TC, FC, CE, and TG Content

RAW264.7 macrophages were seeded in 12-well plates for 24 h and treated with P1 and P2 (10~200 µM) as detailed above. TC and FC were determined using BioVision (Inc., Mountain View, CA) total cholesterol and cholesteryl ester colorimetric assay kit II according to the manufacturer’s instructions and cellular TG content was quantified by a commercially available colorimetric TG assay kit (Biomax, Seoul, Republic of Korea) after the treatment of peptides. The protein concentration of treated cells was determined by the BCA assay. CE amount was calculated by subtracting FC from TC.

### 4.7. Determination of Cholesterol Influx and Efflux

RAW264.7 macrophages were treated with P1 or P2 (10~200 µM) for 1 h followed by 24 h treatment of oxLDL in 96-black well plates, and cholesterol influx and efflux effects were determined using 25-NBD-cholesterol following the protocol in our previous study [25]. To determine cholesterol influx, cells were labeled with 25-NBD cholesterol (5 µg/mL) in serum-free DMEM for 6 h after the 24 h treatment, and the cholesterol content in the cells was measured at excitation (485 nm) and emission (535 nm) wavelengths using a GENios microplate reader (GENios, TECAN, Männedorf, Switzerland).

For cholesterol efflux assessment, treated cells were equilibrated with 25-NBD cholesterol (1 µg/mL) for 6 h. After incubation, the 25-NBD cholesterol-labeled cells were washed with PBS and incubated in DMEM for an additional 6 h. The fluorescence of the cholesterol released into the culture medium was measured as described above. Cholesterol efflux was expressed as the percentage of fluorescence intensity in the medium relative to the total fluorescence (fluorescence intensity of cells + fluorescence intensity of medium).

### 4.8. Determination of TNF-α, IL-1β, IL-6, and PGE_2_ Levels

Macrophages were treated with P1 or P2 (10~200 µM) as detailed above, and the level of inflammatory mediators (TNF-α, IL-1β, IL-6, and PGE_2_) were quantified using BioLegend ELISAMAX™ Deluxe kit, USA, and ELISA kit (Cayman Chem. Co., Ann Arbor, MI, USA) according to the manufacturer’s instructions.

### 4.9. Determination of NO Level

NO level in the culture medium was quantified using the Griess reagent assay. Macrophages were treated with P1 and P2 as detailed above, and NO level in the culture medium was determined following the protocol in our previous study [57]. A 50 μL sample of the culture supernatant was combined with 50 μL of Griess reagent and incubated for 20 min. The absorbance was then measured at 540 nm using a microplate reader. The NO concentration was calculated using a standard curve derived from sodium nitrite.

### 4.10. Western Blot Analysis

Western blot analysis was conducted following a standard protocol. Briefly, whole cell lysates were prepared using RIPA buffer with protease and phosphatase inhibitors (Roche Diagnostics, Seoul, Korea) after treating the cells as previously described. The nuclear fraction was extracted using the NE-PER Nuclear and Cytoplasmic Extraction Reagents kit (NE-PER Nuclear and Cytoplasmic Extraction Reagents, Thermo Scientific) according to the manufacturer′s instructions. Bands were visualized using a chemiluminescence ECL assay kit (Life Technologies, Seoul, Republic of Korea) and imaged with a Davinch-Chemi Imager™ (CAS400SM, Core Bio, Seoul, Korea).

### 4.11. Statistical Analysis

Sigma Plot 12.0 (Systat Software Inc., San Jose, CA, USA) was used to perform a one-way ANOVA and data are presented as means ± SD (*n* = 3). Student’s *t*-test was performed and values with *p* < 0.05 were regarded as statistically significant.

## 5. Conclusions

Multifunctional marine-derived BAPs with anti-inflammatory properties represent an attractive strategy for mitigating foam cell formation preventing atherosclerosis. Hence, in this study, we investigated the foam cell formation inhibitory activity and associated anti-inflammatory activities of two blue mussel-derived peptides PIISVYWK (P1) and FSVVPSPK (P2). P1 and P2 peptides demonstrated promising effects on foam cell formation inhibition and maintaining cholesterol flux processes. They attenuated cholesterol influx, enhanced cholesterol efflux, and led to increased activation of transcription factors PPAR-γ and LXR-α. Moreover, P1 and P2 peptides inhibited foam cell formation in hASMCs. They also demonstrated anti-inflammatory effects by inhibiting the nuclear activation of NF-κB and showed anti-oxidative stress properties through the activation of Nrf2 nuclear translocation. These results suggest that P1 and P2 peptides from blue mussels may offer therapeutic potential in the prevention and treatment of atherosclerosis and related metabolic diseases. However, further in vivo studies are necessary to confirm and expand upon these findings.

## Data Availability

The data presented in this study are available on request from the corresponding authors.

## Abbreviations

BAPs: bioactive peptides, P1: PIISVYWK, P2: FSVVPSPK, CVDs: cardiovascular diseases, CD36: cluster differentiation 36, SR-A1: class A1 scavenger receptor, ABCA-1: ATP-binding cassette transporter A1, ABCG-1: ATP-binding cassette subfamily G member 1, PPAR-γ: peroxisome proliferator-activated receptor-gamma, LXR-α: liver X receptor-alpha, TNF-α: tumor necrosis factor alpha, IL-6: Interleukin 6, IL-1β: interleukin-1β, NO: nitric oxide, PGE_2_: prostaglandin E_2_, iNOS: inducible nitric oxide synthase, COX-2: cyclooxygenase-2, NF-κB: Nuclear factor kappa B, HO-1: heme oxygenase-1, Nrf2: nuclear factor erythroid 2-related factor 2, LDL: low-density lipoproteins, oxLDL: oxidized-low density lipoproteins, BAPs: bio-active peptides, DMEM: Dulbecco′s modified eagle medium, HASMC: human aorta vascular smooth muscle cells, ORO: Oil Red O, TC: total cholesterol, FC: free cholesterol, CE: cholesterol ester, TG: triglycerides, SIM: simvastatin, RSG: rosiglitazone, MDA: Malondialdehyde
